# Thermosensitive Bioadhesive Hydrogels Based on Poly(*N*-isopropylacrilamide) and Poly(methyl vinyl ether-*alt*-maleic anhydride) for the Controlled Release of Metronidazole in the Vaginal Environment

**DOI:** 10.3390/pharmaceutics13081284

**Published:** 2021-08-17

**Authors:** Ana V. Torres-Figueroa, Cinthia J. Pérez-Martínez, J. Carmelo Encinas, Silvia Burruel-Ibarra, María I. Silvas-García, Alejandro M. García Alegría, Teresa del Castillo-Castro

**Affiliations:** 1 Departamento de Investigación en Polímeros y Materiales, Universidad de Sonora, Hermosillo 83000, Mexico; anavaleria.torresf@gmail.com (A.V.T.-F.); carmelo.encinas@unison.mx (J.C.E.); silvia.burruel@unison.mx (S.B.-I.); 2 Departamento de Ciencias Químico Biológicas, Universidad de Sonora, Hermosillo 83000, Mexico; jhovanna.perez@unison.mx (C.J.P.-M.); monserrat.garcia@unison.mx (A.M.G.A.); 3 Departamento de Investigación y Posgrado en Alimentos, Universidad de Sonora, Hermosillo 83000, Mexico; maria.silvas@unison.mx

**Keywords:** nanocomposite hydrogel, thermosensitive hydrogel, bioadhesive hydrogel, controlled drug release, metronidazole

## Abstract

The development of thermosensitive bioadhesive hydrogels as multifunctional platforms for the controlled delivery of microbicides is a valuable contribution for the in situ treatment of vagina infections. In this work, novel semi-interpenetrating network (s-IPN) hydrogels were prepared by the entrapment of linear poly(methyl vinyl ether-*alt*-maleic anhydride) (PVME-MA) chains within crosslinked 3D structures of poly(*N*-isopropylacrylamide) (PNIPAAm). The multifunctional platforms were characterized by Fourier transform infrared spectroscopy, scanning electron microscopy, thermal techniques, rheological analysis, swelling kinetic measurements, and bioadhesion tests on porcine skin. The hydrogels exhibited an interconnected porous structure with defined boundaries. An elastic, solid-like behavior was predominant in all formulations. The swelling kinetics were strongly dependent on temperature (25 °C and 37 °C) and pH (7.4 and 4.5) conditions. The s-IPN with the highest content of PVME-MA displayed a significantly higher detachment force (0.413 ± 0.014 N) than the rest of the systems. The metronidazole loading in the s-IPN improved its bioadhesiveness. In vitro experiments showed a sustained release of the antibiotic molecules from the s-IPN up to 48 h (94%) in a medium simulating vaginal fluid, at 37 °C. The thermosensitive and bioadhesive PNIPAAm/PVME-MA systems showed a promising performance for the controlled release of metronidazole in the vaginal environment.

## 1. Introduction

Temperature-sensitive hydrogels (TSHs) have been well studied as stimuli responsive materials for controlled drug release [1,2]. These systems undergo a sol–gel transformation or a volume phase transition after achieving a critical temperature. The swelling and deswelling behavior of some TSHs can be tailored by the suitable tuning of temperature. This effect is convenient to trigger the release of drugs from the hydrogel under suitable thermal conditions, e.g., at physiological temperature.

The poly(*N*-isopropylacrylamide) (PNIPAAm) is a thermosensitive polymer with a lower critical solution temperature (LCST) of around 32 °C [3]. PNIPAAm-based systems of variable architectures have been prepared for their application in the clinical field including block and grafted copolymers [4], self-assembly conjugates [5], interpenetrating polymer networks (IPNs) [6], and semi-interpenetrating polymer networks (s-IPNs) with crosslinked PNIPAAm [7]. This polymer has been proposed for topical treatments in the vagina [4]. PNIPAAm-based hydrogels go through a volume phase transition by increasing temperature above their LCST, resulting in the removal of large amounts of drug-loaded solution from the materials [8].

The combination of thermo-responsive polymers with bioadhesive materials has potential to improve the efficacy of local treatments. The adhesive interactions with, for example, mucin-coated epithelial surfaces enhance the residence time of dosage forms at the application site, increasing the drug bioavailability [9]. Furthermore, drugs can be successfully delivered to the systemic circulation, e.g., via vaginal mucosa, for the treatment of various diseases like migraine and osteoporosis [10].

Polymers recognized for their intrinsic bioadhesive properties have been combined with the PNIPAAm to design multifunctional materials for biomedical uses. For example, Sosnik et al. synthesized chitosan-g-PNIPAAm micelles for the delivery of hydrophobic drugs via mucosa [11]. These polymeric micelles retained both the intrinsic mucoadhesive nature of chitosan and the thermo-responsive properties of PNIPAAm. Wiltsey et al. developed an adhesive system that supports the tissue repair by mixing an injectable copolymer of PNIPAAm-*g*-chondroitin sulfate with alginate microparticles [12]. The adhesive strength of the scaffold was maximized with the addition of alginate particles to the PNIPAAm copolymer. Klemetsrud et al. found promising mucoadhesive properties for liposomes coated with poly(*N*-isopropylacrylamide-*co*-methacrylic acid) (p(NIPAAM-*co*-MAA)) in simulated conditions of the oral cavity [13]. This result is consistent with the adhesive nature of MAA-based polymers, commercially known as Eudispert [14].

In this regard, the development of bioadhesive drug carriers is an important goal in the treatment of bacterial vaginosis (BV). The topical administration of metronidazole (MTZ) is one of the most common treatments for this disease [15]. The antibiotic is typically marketed in creams, vaginal washing, and suppositories. However, traditional presentations have shown limitations to guarantee the permanence requirements of the drug excipients in the vaginal mucosa due to factors such as the orthostatic posture and vaginal secretions, leading to incomplete therapies or unnecessary waste of the drug [16].

Microbicide-loaded hydrogels have been studied for sustained vaginal drug delivery, since in some clinical trials the maintenance of therapeutic drug concentrations for a prolonged period is essential. Sundara et al. demonstrated the potential application of subtilosin-containing polyethylene glycol hydrogels for BV prophylaxis [17]. The hydrogels displayed an initial rapid-release phase (24 h) followed by a slow, sustained-release phase. In another approach, Malli et al. designed a thermosensitive and mucoadhesive hydrogel based on pluronic^®^ F127, chitosan, and MTZ for the treatment of trichomoniasis, a recurrent vaginal infection [18]. The in situ forming hydrogel restricted the MTZ absorption through vaginal mucosa, prolonging the drug activity against *T. vaginalis*. Recently, Giordani et al. found a suitable performance of sodium hyaluronate networks for the controlled release of chlorhexidine in the vaginal cavity, based on the mechanical resistance of the material, its water uptake ability, mucoadhesion, in vitro drug release behavior, and antimicrobial activity [19].

Overall, the development of mucoadhesive thermosensitive hydrogels as multifunctional platforms for the controlled drug delivery is a valuable contribution to the current therapeutic applications, especially in the treatment of BV.

In this work, novel thermosensitive bioadhesive s-IPNs were prepared by the entrapment of linear poly(methyl vinyl ether-*alt*-maleic anhydride) (PVME-MA) chains within crosslinked structures of PNIPAAm. PVME-MA is an FDA-approved polymer known for its biodegradability, biocompatible properties, and low toxicity [20]. This bioadhesive synthetic copolymer, commercially known as Gantrex, has been used in formulations of dental adhesives [21], as a scaffold for tissue engineering [20], and in microneedle arrays for drug delivery [22]. The multifunctional platforms were characterized by Fourier transform infrared spectroscopy, scanning electron microscopy, thermal techniques, rheological analysis, swelling kinetic measurements, and bioadhesion tests on porcine skin. The loading and in vitro release studies of MTZ from the PNIPAAm/PVME-MA s-IPN in simulated vaginal conditions evidenced the potential of these thermosensitive and bioadhesive materials as drug carriers in the treatment of BV.

## 2. Materials and Methods

### 2.1. Materials

*N*-isopropylacrylamide (NIPAAm) 97%, *N,N*′-methylenebisacrylamide (MBA) 99%, *N,N,N′,N*′-tetramethylethylenediamine (TEMED) 99%, potassium persulfate (KPS) 99%, poly(methyl vinyl ether-*alt*-maleic anhydride) (PVME-MA) 216,000 g mol^−1^, sodium dihydrogen phosphate 99%, disodium hydrogen phosphate 99%, citric acid 99.5%, potassium hydroxide 85%, calcium hydroxide 95%, bovine serum albumin 96%, lactic acid 85%, acetic acid 99.7%, glycerol 86%, urea 98%, glucose anhydrous 96%, and metronidazole (MTZ) 98% were purchased from Sigma-Aldrich. Sodium chloride 99% was obtained from Meyer. All reagents were of analytical grade and used as received without further purification. The aqueous solutions were prepared with deionized water, purified by a Milli-Q Organex system (Millipore, Molsheim, France).

### 2.2. Preparation of s-IPN

**Figure 1:**
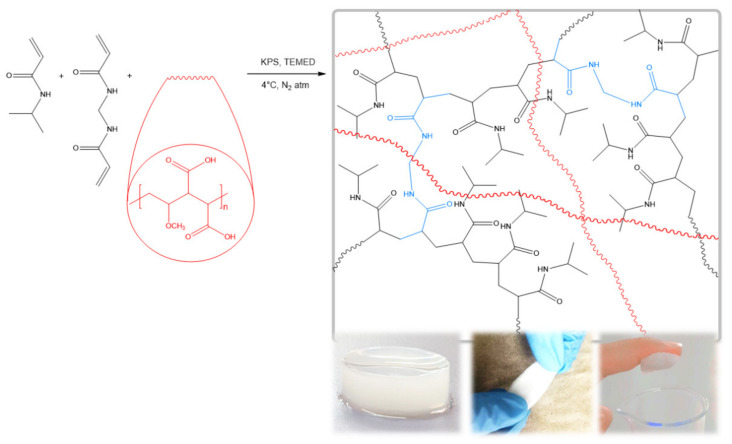
Schematic representation of s-IPN formation. The photographs illustrate, from left to right, the appearance of as-formed composite hydrogels, the ease of handling of dehydrated samples, and the sticky nature of wet material.

The s-IPNs were prepared by adding NIPAAm monomer, MBA, KPS, and TEMED into PVME-MA copolymer solution, as shown in Figure 1. Briefly, NIPAAm 10% solution was prepared under nitrogen atmosphere at 4 °C. The appropriate amount of MBA was added to the former solution in a molar ratio of MBA to NIPAAm of 1:50. A PVME-MA 10% solution, previously hydrolyzed at 60 °C for 6 h, was added to the monomeric solution, keeping the initial conditions. Then, 1 mL of KPS 2% solution was added and the mixture was stirred. Afterwards, 25 μL of TEMED was added with further stirring. Next, the resultant mixture was poured into a cylindrical mold of 25 mm diameter chilled in an ice bath (4 °C). Finally, the hydrogel was removed from the mold, washed with deionized water, and dried by lyophilization.

Neat PNIPAAm hydrogels were also prepared by a similar procedure without the addition of PVME-MA. Table 1 summarizes the volumes used for the preparation of hydrogels.

### 2.3. Characterizations

Fourier transformed infrared spectroscopy (FTIR) spectra were recorded in a Frontier spectrometer (PerkinElmer, Beaconsfield, UK) by the KBr pellet technique.

Scanning electron microscopy (SEM) was used to study the internal morphology of samples. The characterizations were performed using a scanning electron microscope model JEOL JSM-5410LV (JEOL-LTD, Tokyo, Japan) equipped with an INCA system and an X-ray dispersive energy microanalysis detector (Oxford Instruments, Buckinghamshire, UK), operated with an acceleration voltage of 15 kV. The samples were gold sputtered prior to SEM examination.

TGA experiments were carried out under nitrogen flow until 800 °C and a heating rate of 10 °C min^−1^, using a Pyris 1 apparatus (PerkinElmer, Llantrisant, UK).

Rheological measurements were performed in an MCR 502 rheometer (Anton Paar, Graz, Austria) at room temperature, using a parallel plate fixture. The plate diameter was 50 mm. Frequency sweeps were carried out over the range 0.1–100 rad/s under a maximum shear strain of 0.5%. The rheological measurements were performed at least in triplicate for each formulation, and a representative curve of each material was chosen to show the general trend.

Swelling properties were investigated by the gravimetric method at temperatures of 25 and 37 °C. Xerogel samples of known weight (*w*_0_) were immersed in sodium phosphate buffer (pH 7.4, 100 mM) or citrate–phosphate buffer (pH 4.5, 100 mM). At specific times (*t*), the samples were removed from the swelling medium, blotted, weighed (*w_t_*), and placed in the same bath until constant weight was reached. The swelling percent at time *t* was calculated from the following relation:

(1) $$Swelling\;\left(\%\right)=\frac{w_{t}-w_{0}}{w_{0}}\times 100\%$$

### 2.4. Bioadhesion Analysis

In vitro adhesion tests were carried out to measure the bulk adhesive strength of hydrogels using a Texture Analyzer TA.XT Plus (Stable Micro System, Surrey, England). The samples were hydrated with a medium simulating vaginal fluid (MSVF) [23]. The composition of MSVF is shown in Table 2. The analyses were performed at room temperature. Porcine skin was used as a testing surface to mimic human skin tissues.

The porcine skin was obtained from a local market; the subcutaneous fat was removed, and the skin was immediately washed with isopropyl alcohol. The processed porcine tissue was cut into 10 × 10 mm squares, and one piece was attached to the upper aluminum cylinder probe (6 mm diameter) of the texturometer using a cyanoacrylate glue. An equal portion of tissue was fixed on the Peltier plate using the same glue. A hydrogel sample, 10 mm wide, 10 mm long, and 3 mm thick, was placed on the porcine skin glued to Peltier plate. A preload of 1 N was applied for 60 s to the upper side of hydrogel (to be sandwiched between the porcine tissues), and then the upper probe was raised at a constant speed of 2 mm/min. The force required to detach the hydrogel from the tissue surface was determined as the peak value in resultant force–time plot.

The data are presented as mean ± standard deviation. For comparative studies, the data were analyzed by analysis of variance (ANOVA) with an acceptable significance level of *p* < 0.05 using the statistical package NCSS 2007.

### 2.5. Loading and Releasing Studies of MTZ

Each xerogel sample was loaded by sorption in 2 mL of 0.75 wt% MTZ solution, followed by freeze-drying. The experiments of drug release were performed in citrate–phosphate buffer (pH 4.5, 100 mM) and MSVF, at temperatures of 25 and 37 °C. The MTZ-loaded hydrogels were immersed in 50 mL of buffer solution. At specific time intervals, samples of 200 μL were withdrawn and replaced with equal volumes of fresh medium. The MTZ concentration was determined by recording the absorbance at 320 nm in an UV–Vis spectrophotometer model 8453 (Agilent Technologies, Shangai, China), and subsequently interpolating the value in a calibration curve.

## 3. Results and Discussion

### 3.1. FTIR

**Figure 2:**
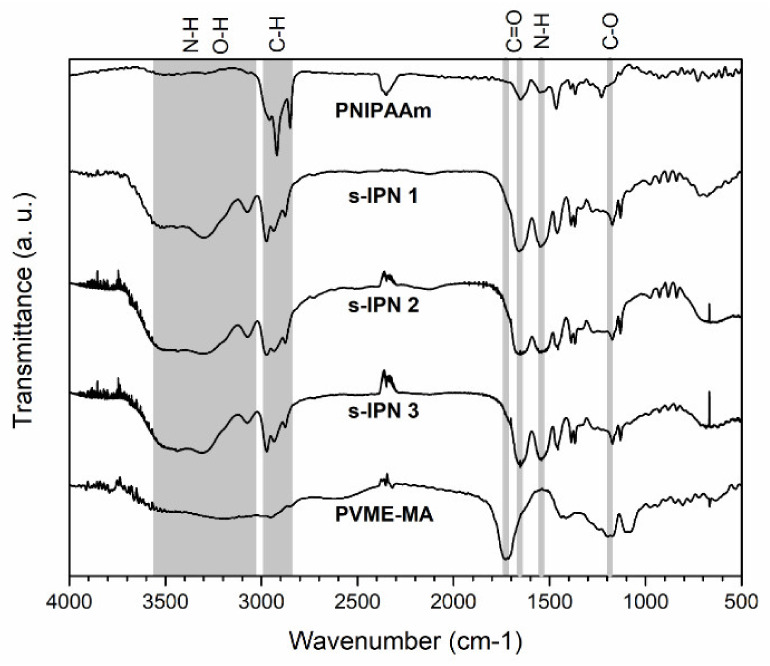
FTIR spectra of PMVE-MA, pristine PNIPAAm hydrogel, and s-IPN samples.

Figure 2 shows the FTIR spectra of the hydrolyzed form of PMVE-MA, neat PNIPAAm hydrogel, and those of s-IPN samples of different compositions.

The spectrum of the hydrolyzed form of copolymer shows a band from 3500 to 3030 cm^−1^ related to stretching vibration of hydroxyl groups (OH) of carboxyl moieties (hydrolyzed MA unit). This broad band partially overlaps with absorption bands between 2980 and 2840 cm^−1^, which are assigned to symmetrical and asymmetrical C–H stretching vibrations of CH (hydrolyzed MA unit), CH_2_, and CH_3_ (MVE unit) groups [24]. The sharp band at 1728 cm^−1^ is attributed to stretching vibration of the carbonyl group (C=O) (hydrolyzed MA unit). The signal at 1173 cm^−1^ is assigned to stretching vibration of the ether group (C–O–C) (MVE unit) [24].

The spectrum of PNIPAAm hydrogel displays the typical absorptions of its polymer precursor. The broad band in the spectral region from 3500 to 3160 cm^−1^ is assigned to N–H stretching of a secondary amide. Strong bands due to C-H vibrations of CH, CH_2_, and CH_3_ groups are observable in the range of 3000 to 2837 cm^−1^. The C=O group gives rise to the band at 1651 cm^−1^ (amide I), while the N–H in-plane bending peak appears at 1549 cm^−1^ (amide II). The band at 1465 cm^−1^ and the doublet peak between 1397 and 1355 cm^−1^ are attributed to the bending of C-H bond and deformation of the isopropyl group (C(CH_3_)_2_), respectively [25].

The characteristic individual absorptions of PVME-MA and PNIPAAm polymers are distinguished in the spectra of s-IPN samples; however, spectral shifts are also detected. The bands due to O–H (hydrolyzed MA unit of PVME-MA) and N–H (PNIPAAm) vibrations overlap in the region from 3600 to 3150 cm^−1^, appearing as a well-resolved contribution at 3073 cm^−1^ in all three spectra. This lower wavenumber signal can be related to the hydroxyl groups of the interpenetrated polymer, and also to the amine groups of the polymer network involved in H-bonding interactions. The relative intensity of C=O band increases in s-IPN spectra with respect to the PNIPAAm spectrum as result of the copolymer contribution (hydrolyzed MA unit). Moreover, this signal shifts to lower wavenumbers (1657 cm^−1^) as compared to its position in the PVME-MA spectrum. This effect is consistent with the participation of the carbonyl group of the copolymer in H–bonding interactions [26]. The presence of PVME-MA within the crosslinked PNIPAAm chains is confirmed by the ether peak (MVE unit) at 1173 cm^−1^ in all three s-IPN spectra.

### 3.2. SEM Analysis

**Figure 3:**
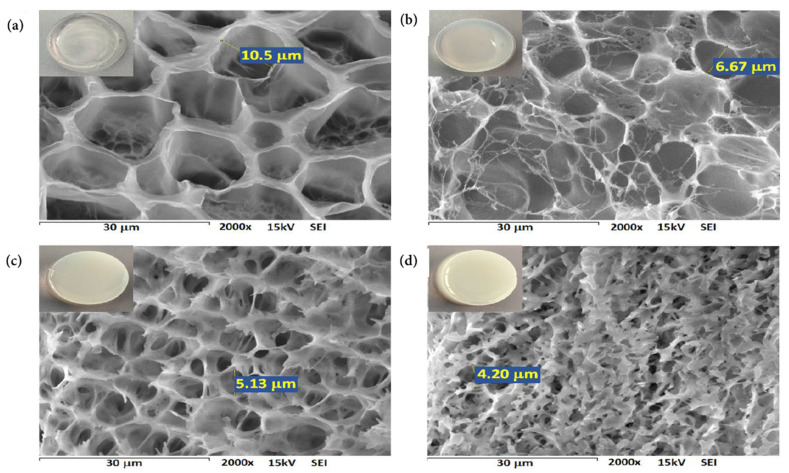
SEM micrographs of cross sections of PNIPAAm (**a**), s-IPN 1 (**b**), s-IPN 2 (**c**), and s-IPN 3 (**d**) hydrogels.

Figure 3 shows the exterior appearance and the internal microstructure of single PNIPAAm network hydrogel and s-IPN samples. The s-IPN showed a whitish appearance, in contrast with the translucent aspect of the PNIPAAm hydrogel. All samples displayed an internal porous structure with defined boundaries. The presence of smaller pores within larger cavities evidences the pore interconnection, which is a desirable feature for capillary transport of biological fluids and drug solutions through the polymer matrix [27,28].

Different pore sizes were detected among hydrogels. The pore dimensions of all s-IPN samples are smaller than the pore size of the PNIPAAm sample (~10 μm). Moreover, the increase in PVME-MA content decreased the pore dimensions of composite networks. In this work, the amounts of NIPAAm monomer and MBA crosslinker were decreased from PNIPAAm to s-IPN 3 samples, so the proportion of crosslinked networks should also be reduced. The denser structures of s-IPN could be related to the H-bonding interaction between the PNIPAAm network and the entrapped PVME-MA copolymer, which is consistent with the previously shown FTIR results. The non-covalent interactions favor the homogeneous distribution of the copolymer within the free volume of the PNIPAAm network, reducing the sizes of pores left by the ice crystal sublimation during the freeze-drying of materials. The reduction in internal pore sizes of s-IPN with the increase in interpenetrated polymer content is closely related to non-covalent interactions [29,30].

### 3.3. TGA Analysis

**Figure 4:**
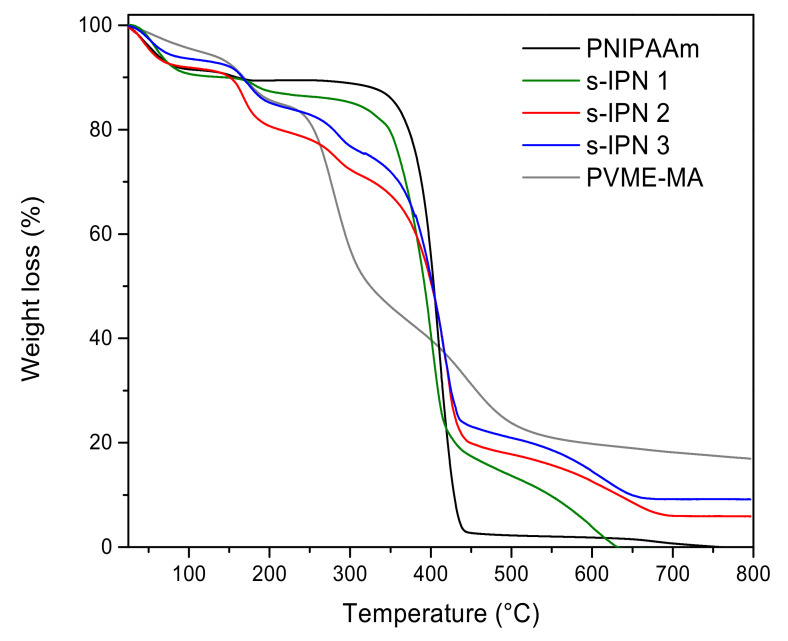
Thermograms of neat PNIPAAm hydrogel, PMVE-MA, and s-IPN samples.

Figure 4 shows the thermograms of hydrolyzed PMVE-MA, neat PNIPAAm hydrogel, and those of s-IPN samples of different compositions. Table 3 summarizes the temperatures of the maximum rate of weight loss (*T_max_*) of each weight loss step for different materials.

All samples lost mass at temperatures below 100 °C, which was associated with the evaporation of residual moisture. The PVME-MA exhibited a multi-step degradation process. The degradation step with *T_max_* at 170 °C was related to the dehydration of carboxyl groups, while final steps were attributed to the degradation of the copolymer backbone [31]. Conversely, the thermal degradation of PNIPAAm hydrogel occurred in a single weight loss step, in accordance with previous findings [32].

The mass loss step at *T_max_*~170 °C, associated with the formation of carboxylic acid anhydride groups in PVME-MA, was detected in thermograms of s-IPN samples, confirming the entrapment of copolymer within the PNIPAAm network. Further degradation steps of individual polymers overlap in the interval from 280 to 600 °C.

### 3.4. Rheological Measurements

**Figure 5:**
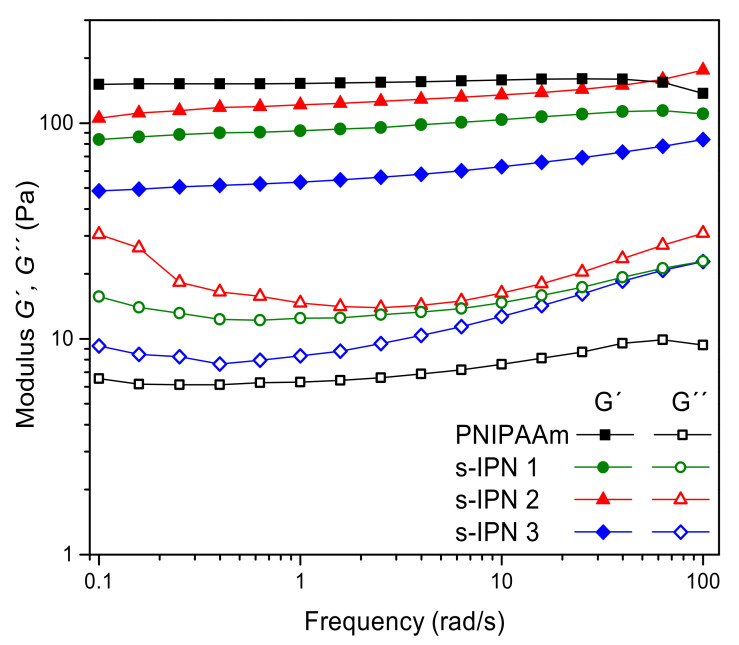
Rheological measurements of neat PNIPAAm hydrogel and s-IPN samples.

The viscoelastic properties of neat PNIPAAm hydrogel and those of s-IPN samples in similar hydration conditions were analyzed by rheological measurements. Storage (G′) and loss (G″) dynamic moduli of hydrogels as a function of frequency are shown in Figure 5.

The frequency dependence of G′ and G″ for the PNIPAAm hydrogel showed a “true gel” type behavior: a slight variation in dynamic moduli with the frequency was observed and the mechanical loss tangent (tanδ = G″/G′) was smaller than 0.1 (from 0.04 to 0.07) in the whole frequency range [33]. A similar elastic, solid-like nature of MBA-crosslinked PNIPAAm hydrogels has been reported elsewhere [34].

Rheological properties were expected to change with the decrease in PNIPAAm content, the polymer that forms the interconnected elastic framework. For the s-IPN samples, a greater frequency dependence of G″ and smaller separation between the two moduli were observed as compared to the neat PNIPAAm hydrogel. The values of tanδ were found to be between 0.11 and 0.29, which is associated with a “weak gel” network [33,35]. This viscoelastic condition can favor the flow of hydrogel within some cavities of the human body, thereby minimizing the disturbance of the biological environment and increasing its capacity to make intimate contact with the tissue substrate [36]. Moreover, it should be noticed that the G′ continued to be almost independent of frequency, and G′ values were found larger than G” in all s-IPNs, indicating that the solid-like character remained predominant over the liquid-like response, as in single PNIPAAm hydrogel.

### 3.5. Swelling Kinetic Measurements

**Figure 6:**
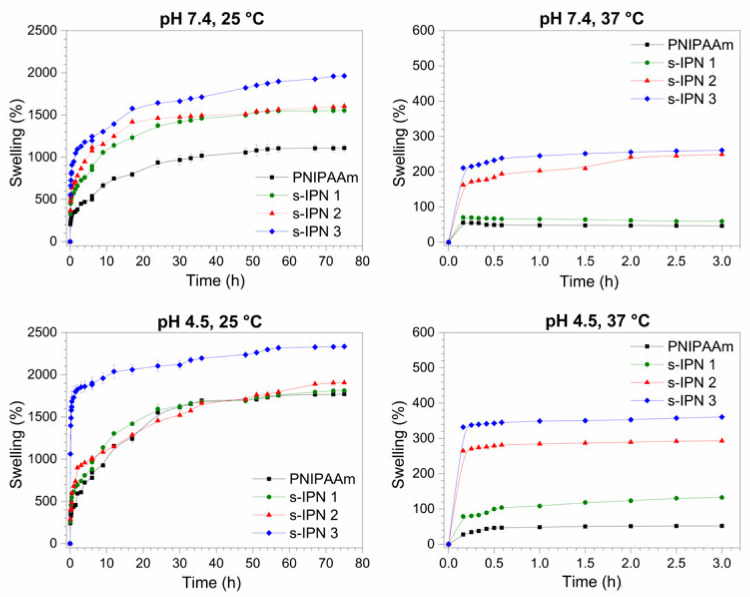
Swelling kinetics of the neat PNIPAAm hydrogel and those of s-IPN samples in buffer media pH 7.4 and pH 4.5, at temperatures of 25 and 37 °C. The experimental points were joined using a spline line function.

Figure 6 shows the swelling behavior of neat PNIPAAm hydrogel and that of s-IPN samples. At fixed conditions of pH and temperature, higher swelling degrees at equilibrium were observed for s-IPNs as compared to the PNIPAAm hydrogel. Moreover, swelling levels increased with the PVME-MA content, which was associated with the contribution of hydrophilic copolymer.

The thermo-responsive behavior, owing to the crosslinked PNIPAAm, was observed in all hydrogels at both pH values. At 25 °C, the hydrogels reached swelling ratios higher than 1000% at pH 7.4 and 4.5. The tendency to form hydrogen bonds (H-bonds) between the hydrophilic groups of PNIPAAm and water molecules prevails at temperatures below the phase transition temperature, thereby promoting water uptake into the hydrogel network. The swelling capabilities of all hydrogels drastically decreased at 37 °C, exhibiting equilibrium swelling ratios lower than 400% at both pH values. The time to reach the swelling equilibrium was also shortened to around 1 h in all samples. The hydrophobic interactions between the PNIPAAm moieties became prominent at temperatures above the transition temperature, leading to a dramatic decrease in the swelling capabilities of hydrogels.

Besides the thermosensitive behavior, the hydrogels showed pH-responsive properties, which were more pronounced for s-IPN samples. The swelling percent at equilibrium of the three composite hydrogels increased at pH 4.5 with respect to pH 7.4 at both temperatures. The carboxyl side groups of PVME-MA (pKa 6.5 and 3.5 [37]) are fully deprotonated and half deprotonated at pH 7.4 and 4.5, respectively. The net charge of interpenetrated copolymer affects its interactions: PVME-MA-PVME-MA and PVME-MA-PNIPAAm network. Ionized donor or acceptor groups can form short charge-assisted H-bonds that are typically stronger than ones between neutral groups. These strengthened H–bonds play a key functional role in several proteins [38]. At pH 7.4, both charged –COO– moieties of PVME-MA are able to form strong H–bonds with the H–donor–CONH– group of the crosslinked PNIPAAm. This network reinforcement can restrict the motion of the PNIPAAm segments, limiting the water uptake at physiological pH. On the other hand, at pH 4.5, short COOH– –OOC intramolecular H–bonds in the PVME–MA would be favored over the strengthening of the PNIPAAm network. This effect can explain the enhancement of swelling capabilities of s-IPNs in an acidic environment with respect to their behavior at pH 7.4.

**Figure 7:**
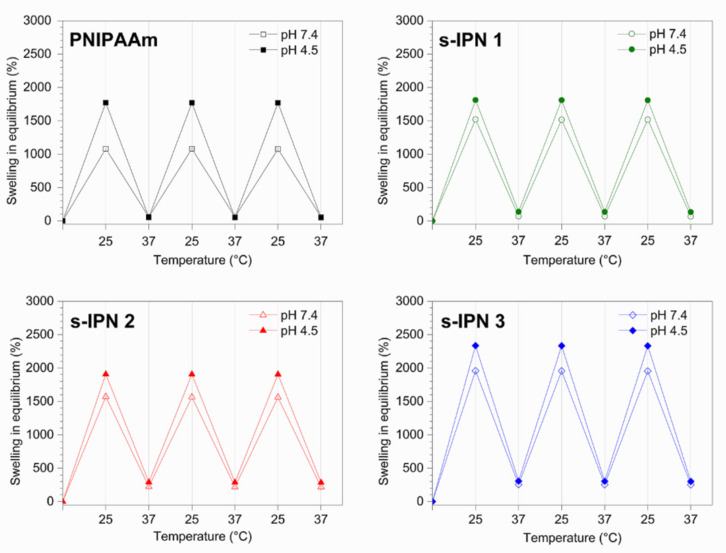
Cycles of swelling/shrinking of the neat PNIPAAm hydrogel and s-IPN samples, under alternating temperatures of 25/37 °C. Error bars are included in the graphs.

The reversibility of thermal-induced swelling/shrinking behavior of hydrogels was assessed by varying the temperature between 25 and 37 °C, in pH 7.4 and 4.5 media. The hydrogels were allowed to reach the equilibrium condition at each temperature. Figure 7 shows the swelling degree of the different samples under three consecutive cycles of temperature change. The variations in swelling degree are fully reversible within the tested conditions, i.e., values at 25 and 37 °C stay the same from one cycle to another.

### 3.6. Bioadhesive Analysis

Bioadhesion refers to the interaction between synthetic or natural macromolecules and biological tissues [39]. Hydrogels with a higher content of the adhesive copolymer (s-IPN 2 and s-IPN 3) were selected to evaluate their bioadhesive behavior.

**Figure 8:**
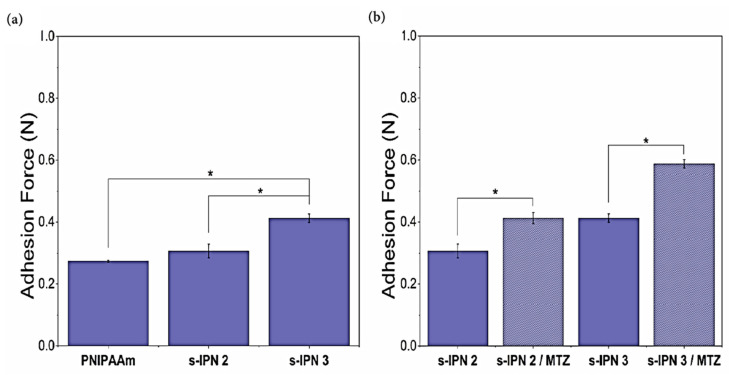
Bioadhesive properties of PNIPAAm, s-IPN 2, and s-IPN 3 samples (**a**), and those of s-IPN 2, s-IPN 3 with and without MTZ (**b**). Data are shown as the mean ± SD from three independent replicates. Statistical significance (* *p* < 0.05) was determined by ANOVA with a Tukey post hoc test.

Figure 8a shows the detachment force obtained from adhesion tests for PNIPAAm, s-IPN 2, and s-IPN 3 samples hydrated with MSVF. An analysis of variance (ANOVA) was conducted between the three groups with freedom degrees (*FD*) of 8 and *n* = 3. Since the calculated *F* value and the critical *F* value were 66.5544 and 5.1432, respectively, the detachment forces were significantly different between samples (*p* = 0.00008). The Tukey post hoc test revealed that the adhesion force of PNIPAAm hydrogel (0.274 ± 0.003 N) was not significantly different from the slightly higher value of the s-IPN 2 hydrogel (0.308 ± 0.022 N) (*p* > 0.05). However, the s-IPN 3 sample, with the highest content of PVME-MA, displayed a significant higher detachment force (0.413 ± 0.014 N) than the rest of the hydrogel systems (*p* < 0.05). As mentioned before, the PVME-MA has been recognized for its bioadhesiveness. The carboxyl group content and the polyanionic nature of the copolymer at the MSVF pH condition (pH 4.6) allow forming hydrogen bonds and/or electrostatic interactions between proteins on the surfaces of porcine skin tissue and the polymeric chains of the hydrogel [40,41].

Figure 8b shows the adhesive properties of s-IPN 2 and s-IPN 3, with and without MTZ. ANOVA compares two groups, s-IPN 2/s-IPN 2-MTZ and s-IPN 3/s-IPN 3-MTZ, with *FD* = 5 and *n* = 3. The adhesion force of s-IPN 2-MTZ was significantly higher than that of s-IPN 2 (calculated *F* = 39.7552, critical *F* = 7.7086, *p* = 0.0032). The same trend was followed by the second group; the adhesive properties of s-IPN 3-MTZ were superior with respect to the s-IPN 3 sample (calculated *F* = 237.6187, critical *F* = 7.7086, *p* = 0.0001). The Tukey post hoc analysis indicated significant differences for s-IPN 2/s-IPN 2-MTZ samples (*p* < 0.05), and also for s-IPN 3/s-IPN 3-MTZ hydrogels (*p* < 0.05). Hence, the MTZ loading in the s-IPN samples increased the adhesion force for both formulations. This feature was associated with the hydroxyl group contribution of the MTZ that increased the probability of H-bonding interactions between the skin tissue surface and the MTZ-containing hydrogels. Wróblewska et al. [42] found that MTZ-containing gel formulations (bigels, hydrogels, oleogels) exhibit higher adhesive forces on porcine buccal mucosa than the formulations without MTZ.

The bioadhesive performance of MTZ-loaded formulations s-IPN 2 (0.412 ± 0.018 N) and s-IPN 3 (0.587 ± 0.013 N) was superior to that obtained by Perioli et al. [43] for the market product Zidoval^®^ (bioadhesive force of 0.060 N), using porcine vaginal mucosa tissues of 2 × 2 cm. These authors reported mucoadhesive forces up to 0.196 ± 0.017 N for chitosan-based gels intended for MTZ administration.

### 3.7. Studies of MTZ Release

Taking advantage of their high swelling degrees at 25 °C, the dry samples of s-IPN 2 and s-IPN 3 were loaded by sorption in the drug solution at this temperature, followed by lyophilization.

**Figure 9:**
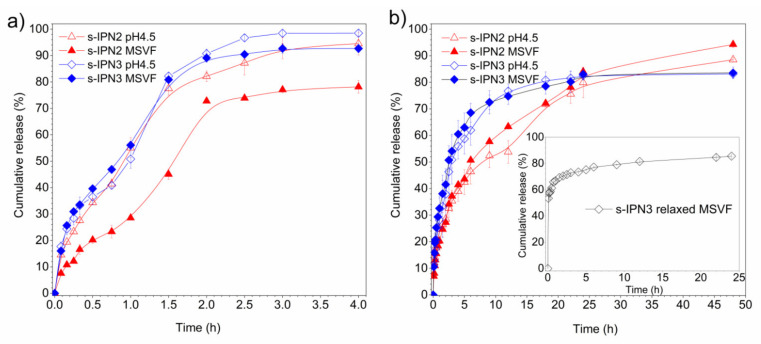
Release kinetics of MTZ from s-IPN 2 and s-IPN 3 in buffer pH 4.5 and MSVF media, at 25 °C (**a**) and 37 °C (**b**). Inset (**b**) shows release profiles from s-IPN 3 in an initial relaxed state. The experimental points were joined using a spline line function.

Figure 9a displays the MTZ release profiles from s-IPN 2 and s-IPN 3 in citrate–phosphate buffer pH 4.5 and MSVF as release media, at 25 °C. Both hydrogels showed a similar release profile, achieving the maximum percentage of cumulative release in a short period of 3 h.

Contrary to the quick delivery observed at 25 °C, the release profiles from initially dehydrated s-IPN 2 and s-IPN 3 samples at 37 °C were prolonged up to 48 h and 24 h, respectively, as shown in Figure 9b. This time shift of the release profiles is in accordance with the temperature-dependent swelling behavior of hydrogels (Figure 6). The low swelling level of hydrogels at 37 °C leads to slow transport of the drug through the crosslinked matrix to the external medium [44,45]. Moreover, the drug can be released from the hydrogels at 37 °C without excessive enlargement of their volume dimensions as compared to the hydrogel expansion that occurs at 25 °C.

Both hydrogels displayed an initial burst release within the first 10 h at physiological temperature, followed by a sustained release stage. This sustained release can improve the bioavailability of the drug during vaginosis treatments [18,46]. It also should be highlighted that the MTZ was consistently released from s-IPN 2 and s-IPN 3 in FVS at 37 °C, reaching 94% and 83% of cumulative amounts, respectively, at equilibrium.

Figure 9b (inset) includes the MTZ release profiles from s-IPN 3 in a relaxed state, i.e., immediately after being loaded with the drug, without the drying by lyophilization. Differences in the release kinetic were observed with respect to the delivery from the initially dehydrated s-IPN 3 sample. The release from the relaxed state of hydrogel exhibited a strong burst profile, since around 61% of the encapsulated drug was delivered within the first 30 min. The preexistence of polymer chain relaxations at 25 °C, followed by the immersion of the drug-loaded hydrogel into a 37 °C warmed release medium, leads to the volume contraction of material and a rapid expulsion of the inner drug solution. This delivery strategy can be useful when high doses of the drug must be locally attained at the target site in short periods.

## 4. Conclusions

Thermosensitive and bioadhesive s-IPNs were successfully prepared by the entrapment of PVME-MA chains within a chemically crosslinked PNIPAAm structure. Hydrogen bonding interactions were formed between both polymers as confirmed by FTIR analysis. Samples of different PVME-MA content exhibited an interconnected porous structure, which favors the inward or outward movement of drug-loaded molecules in the hydrogels. Despite the solid-like behavior that prevailed in all formulations, the increase in the PVME-MA/PNIPAAm ratio caused the softening of hydrogels, improving their ability to make effective contact with the tissue substrates. Swelling levels increased with the PVME-MA content, due to the hydrophilic nature of copolymer. Moreover, the equilibrium swelling of s-IPN depends on temperature (25 and 37 °C) and pH (7.4 and 4.5), owing to the LCST behavior of the PNIPAAm and ionizable carboxyl groups of the PVME-MA, respectively. The temperature-dependent swelling kinetics of s-IPN 2 and s-IPN 3 formulations allowed the loading of these materials with therapeutic amounts of MTZ at 25 °C, due to their high swelling capacities at this temperature. The hydrogels showed a sustained drug delivery at 37 °C in a simulated vaginal environment because of their low swelling capacities in these release conditions. The controlled delivery of the antibiotic is a desired feature for some local treatments of persistent bacterial infections. A high PVME-MA content along with the MTZ loading into hydrogels promoted the molecular interaction of material with porcine tissues, thereby enhancing its bioadhesiveness. The bioadhesive properties of hydrogels can prolong the residence time of the drug in the vagina, thereby improving its therapeutic efficacy against bacterial vaginosis. The attractive properties of PVME-MA/PNIPAAm platforms render them suitable for a range of pharmaceutical and biomedical applications, in particular for the treatment of vaginal infections.

## Figures and Tables

**Table 1:** Feed solution volumes in the preparation of hydrogels.

Code	NIPAAm/MBA (mL)	PMVE-*alt*-MA (mL)
PNIPAAm	2	0
s-IPN 1	1.60	0.40
s-IPN 2	1.46	0.54
s-IPN 3	1.34	0.66

**Table 2:** Composition of MSVF.

Composition in Water	g·L^−1^
Sodium chloride	3.51
Potassium hydroxide	1.40
Calcium hydroxide	0.22
Bovine serum albumin	0.02
Lactic acid	2.00
Acetic acid	1.00
Glycerol	0.16
Urea	0.40
Glucose	5.00

**Table 3:** *T_max_* for PNIPAAm hydrogel, PMVE-MA, and s-IPN samples.

Sample	*T_max_* (°C)
PNIPAAm	410.29
s-IPN 1	173.36, 395.77, 596.5
s-IPN 2	168.35, 282.6, 419.63, 622.56
s-IPN 3	173.59, 284.69, 415.24, 612.47
PVME-MA	170.31, 280.86, 444.85

## Data Availability

The data presented in this study are available on request from the corresponding author.
